# Epigenetic Alterations and Exposure to Air Pollutants: Protocol for a Birth Cohort Study to Evaluate the Association Between Adverse Birth Outcomes and Global DNA Methylation

**DOI:** 10.2196/resprot.7114

**Published:** 2017-02-23

**Authors:** Zhila Maghbooli, Arash Hossein-Nezhad, Majid Ramezani, Syamak Moattari

**Affiliations:** ^1^ Osteoporosis Research Center Endocrinology and Metabolism Research Institute of Tehran University of Medical Sciences Tehran Islamic Republic Of Iran; ^2^ Department of Medicine Section of Endocrinology, Nutrition, and Diabetes, Vitamin D, Skin and Bone Research Laboratory Boston University Medical Center Boston, MA United States; ^3^ Department of Endocrinology Baghiatallah University of Medical Sciences Tehran Islamic Republic Of Iran; ^4^ Health Science Department Worcester State University Worcester, MA United States

**Keywords:** epigenomics, DNA methylation, air pollutants, pregnancy, adverse birth outcomes, placenta

## Abstract

**Background:**

Prenatal exposure to air pollutants can increase the risk of adverse birth outcomes and susceptibility to a number of complex disorders later in life. Despite this general understanding, the molecular and cellular responses to air pollution exposure during early life are not completely clear.

**Objective:**

The aims of this study are to test the association between air pollution and adverse pregnancy outcomes, and to determine whether the levels of maternal and cord blood and of placental DNA methylation during pregnancy predict adverse birth outcomes in polluted areas.

**Methods:**

This is a birth cohort study. We will enroll pregnant healthy women attending prenatal care clinics in Tehran, Iran, who are resident in selected polluted and unpolluted regions before the 14th week of pregnancy. We will calculate the regional background levels of fine particulate matter (particles with a diameter between 2.5 and 10 μm) and nitrogen dioxide for all regions of by using data from the Tehran Air Quality Control Company. Then, we will select 2 regions as the polluted and unpolluted areas of interest. Healthy mothers living in the selected polluted and non polluted regions will be enrolled in this study. A maternal health history questionnaire will be completed at each trimester. During the first and second trimester, we will draw mothers’ blood for biochemical and DNA methylation analyses. At the time of delivery time, we will collect maternal and cord blood for biochemical, gene expression, and DNA methylation analyses. We will also record birth outcomes (the newborn’s sex, birth date, birth weight and length, gestational age, Apgar score, and level of neonatal care required).

**Results:**

The project was funded in March 2016 and enrollment will be completed in August 2017. Data analysis is under way, and the first results are expected to be submitted for publication in November 2017.

**Conclusions:**

We supposed that prenatal exposures to air pollutants can influence fetal reprogramming by epigenetic modifications such as DNA methylation. This could explain the association between air pollution and adverse pregnancy outcomes.

## Introduction

Air pollution, the most pervasive environmental concern, is estimated to cause around 800,000 deaths every year worldwide. Among air pollutants, fine particulate matter is known as a possible cause or exacerbator of diseases [1]. Previous studies showed significant associations between fine particulate matter (particles with a diameter between 2.5 [PM2.5] and 10 μm [PM10]) and mortality from complex disorders such as cardiovascular disease, cardiopulmonary disease, and lung cancer [2].

The highest number of estimated annual premature deaths due to fine particulate matter occurs in the developing countries of Asia [3]. In most Asian cities, sulfur dioxide, nitrogen dioxide, PM2.5 and PM10 levels are above the World Health Organization (WHO) guidelines [3]. Although Tehran, Iran, is rated as one of the world’s most polluted cities, there are few reports on this matter [4,5]. Naddafi et al reported an annual average of 71 μg/m^3^, which is 4.5 times the values recommended by the WHO [4]. Based on a WHO estimation, the average urban PM10 concentration in Iran is 68 µg/m^3^, about 3.4 times higher than the WHO’s air quality guidelines, which is estimated to cause about 9100 deaths per year [6].

Evidence shows that the elderly, children, and pregnant women appear to be more susceptible than the general population to the adverse health effects of air pollution, although people of all age groups are affected by air pollution [7-12]. In addition, growing evidence has been reported of the impact of environmental pollution on adverse birth outcomes [13-15]. Birth outcomes are important for public health policy, because health in early life is crucial for health later in life. Based on a life course approach, most epidemiologic research on chronic diseases has demonstrated that intrauterine and early life conditions significantly affect the occurrence of complex disorders that are of interest to public health [16,17].

Low birth weight, intrauterine growth retardation, and impaired growth in the early years of life are known to increase mortality and morbidity in childhood and the susceptibility of an individual to several complex disorders later in life, such as hypertension, coronary heart disease, and diabetes [15,18]. Despite this general understanding, the biological interactions responsible for impaired development and adverse birth outcomes are not completely clear.

The underlying mechanisms by which air pollutants may induce adverse birth outcomes are not clear. The hypothesis of fetal programming could explain some part of this interaction [19]. This hypothesis states that exposure to endogenous or exogenous factors during a sensitive period can lead to responses at a molecular and cellular level. However, environmental influences on metabolism could persist even under normal conditions or in the absence of stimulating factors [19]. Furthermore, long-term or permanent alterations in the function of target cells can lead to an increased risk for adult-onset diseases such as type 2 diabetes mellitus, hypertension, cardiovascular disease, and cancer [20-22]. The underlying biological mechanisms of fetal programming can be explained by epigenetic modifications such as DNA methylation [23-25], which is one important regulatory mechanism in cell development and differentiation [26,27]. It seems that maternal exposure to air pollutants is associated with an epigenetic modification such as DNA methylation. DNA methylation of cytosine residues is a heritable epigenetic modification that can maintain specific gene expression patterns in different cell types. Alterations in DNA methylation due to metabolic exposure during gestation or after birth may increase the susceptibility of an individual to complex disorders such as cancer and metabolic disorders later in life [28-30].

We systematically searched PubMed and SCOPUS up to December 1, 2016 for literature addressing adverse pregnancy outcomes (infant mortality, postneonatal mortality, birth weight, intrauterine growth retardation, premature birth, birth outcomes, and fetal development), pollution, and DNA methylation. This systematic search identified a few studies that assessed air pollution’s effect on global and gene-specific methylation [31-36]; 2 studies focused on the association of DNA methylation of repetitive elements and global DNA methylation of placental tissue with air pollution in early life [32,36].

An environmental inputs birth cohort study [31] showed that “epigenetic modifications in the mitochondrial genome, especially in the MT-RNR1 region, substantially mediate the association between PM2.5 exposure during gestation and placental mtDNA content, which could reflect signs of mitophagy and mitochondrial death.”

Another study [32] showed “a lower degree of placental global DNA methylation in association with exposure to particulate air pollution during early pregnancy”. It seems that exposure to particulate matter during fetal development can lead to alterations in genomic DNA methylation and affect gene-specific DNA methylation and gene expression patterns during this crucial time. Consistent with this hypothesis, recent evidence from both human subjects and animal models has indicated that exposure to airborne particulate matter is associated with changes in DNA methylation patterns. Alterations of DNA methylation patterns are postulated to modulate immune responses and regulate inflammatory genes in response to inhalation of particulate matter.

Among intracellular pathways, the glutathione pathway’s role in the lung is to defend the airway epithelium from damage in response to oxidants and inflammation [37]. Glutathione is a tripeptide, γ-glutamyl-cysteinyl-glycine (GSH), and is defined as the “body’s master antioxidant” [37]. At the molecular level, nucleotide variation in the glutathione gene has been associated with differences in susceptibility to adverse effects of air pollutants on lung function and growth [38]. Emerging evidence has shown that *S*-adenosylmethionine (SAMe) increases cellular glutathione content and has an important role in the methylation cycle [39,40]. SAMe is the main cosubstrate involved in methyl group transfers in the methylation cycle.

However, it is important to assess whether there is an association between air pollution and adverse birth outcomes, how it is modulated by alteration of genomic DNA methylation in the fetus and placental tissue, and how the adverse effects of air pollution on birth outcomes can be reduced by intervention strategies.

### Study Objective

The primary objective of the study is to compare the incidence of adverse birth outcomes in a polluted urban area with that in an unpolluted urban area.

The secondary objective is to investigate the association between adverse birth outcomes and global changes in fetal and maternal DNA methylation.

In addition, we aim to determine the association between gene expression of GSH and alteration of global DNA methylation.

## Methods

### Study Design

This is a birth cohort study designed by the Endocrinology and Metabolism Research Institute of Tehran University of Medical Sciences. The research has been supported by the National Institute for Medical Research Development of Iran (grant no. 940173).

### Study Population

In our birth cohort study, we will enroll pregnant women attending prenatal care clinics in two regions in Tehran, Iran: the most polluted and the least polluted. The study population is the group of eligible mothers living in these two regions who agree to participate in this study. The inclusion criteria are as follows: (1) resident in the selected polluted and unpolluted regions, (2) stay-at-home mothers (3) presenting to the clinics for prenatal care (4) before the 14th week of pregnancy who are (5) healthy, (6) able to read, write, and understand Persian at a middle school level and (7) willing to participate in follow-up visits. The exclusion criteria are as follows: (1) smoking mothers or living with a smoker, (2) having a history of chronic disorders, including heart disorders, hypertension, diabetes, hyperthyroidism, cancer, or autoimmune disorders such as lupus, (3) taking any kind of corticosteroids, or hyperglycemia or hypertension medications, (4) regularly taking methyl donors such as folate or vitamin B_12_ before pregnancy, or (5) having a multiple pregnancy. If a mother moves out the selected region, is employed, or travels between polluted and unpolluted regions regularly, she will be excluded from the study. The participants in the two regions (polluted and unpolluted) will be matched by age, pregestational body mass index, and parity.

**Figure 1:**
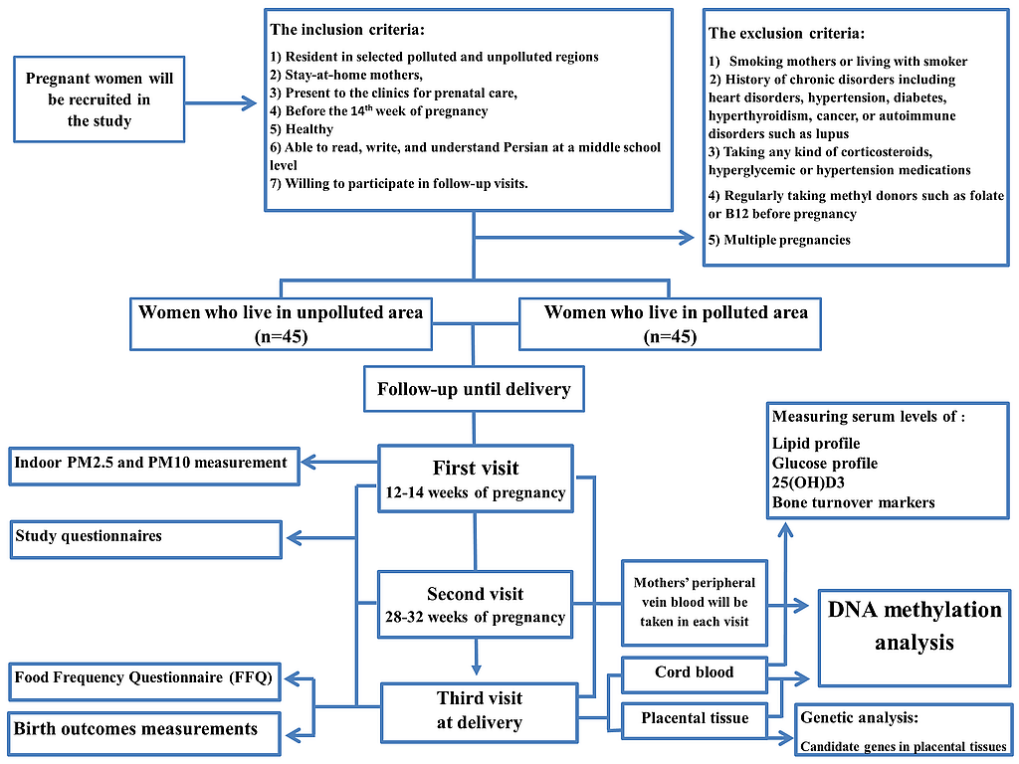
Study design. Flow diagram of selection of pregnant women living in polluted and unpolluted regions of Tehran. PM2.5: particulate matter with particle diameter 2.5 μm; PM10: particulate matter with particle diameter 10 μm.

Written informed consent will be obtained from all study participants in accordance with procedures approved by the Ethical Committee of the National Institute for Medical Research Development (IR.NIMAD.REC.1394.018) of Iran. Consent is attained by a research midwife or a doctor who is not directly involved in the routine perinatal care of the women. A participant may subsequently decide to withdraw from the study at any time without prejudice to their future care. In this study, 40 participants would be required in each group to have 90% power to detect a difference of 4% at global DNA methylation levels between the 2 groups. We anticipate recruiting 80 participants to investigate the role of indoor and outdoor air pollution on global DNA methylation levels of maternal and cord blood and placental tissue. We estimate that the dropout rate during the study will be 10%, so a total of 90 pregnant women will be needed for the duration of the study with a minimum of 45 participants in each subgroup (Figure 1).

### Exposure Measurement

We will calculate the regional background levels of PM10, PM2.5, and nitrogen dioxide for each mother’s home address. The values of air pollutants will be obtained from the Tehran Air Quality Control Company in 4×4 km grids.

To explore the potential effect of exposures during pregnancy, we will calculate regional PM10 and PM2.5 concentrations (micrograms per cubic meter) during various times: the mean levels at 1 week before delivery, during the last month of pregnancy, and for each of the 3 trimesters of pregnancy. We will also calculate the exposure during the whole pregnancy.

To reduce bias due to exposure misclassification, we plan to measure the PM2.5 and PM10, as individual levels, manually by using Dylos DC1100 air quality monitors (Dylos Corporation, Riverside, CA, USA) in each participant’s address (indoor). Also, we will collect drinking water to measure contaminants including hardness, nitrite, and nitrate.

### Data Collection in Each Area

#### First and Second Visits

At the first visit (12-14 weeks of pregnancy), we will complete study questionnaires with the participants to provide detailed information on place of residence, socioeconomic status, sleep habits, smoking status, health status, medical history, and previous pregnancy history. At the second visit (28-32 weeks of pregnancy), a maternal health history questionnaire will be completed to record what medicines, herbs, or vitamins the mother is taking and any adverse events and health problems experienced. We will take the mothers’ peripheral venous blood after an overnight fast of 10-14 hours for biochemical, DNA methylation, and gene expression analyses at the first and second visits.

#### Third Visit (Delivery Time)

We will follow-up participants monthly until delivery. A food frequency questionnaire will be completed to calculate nutrient elements taken in. At the delivery time, we will collect maternal and cord blood for biochemical, gene expression, and DNA methylation analyses. We will also obtain samples of placental tissue for gene expression and DNA methylation analyses.

#### Perinatal Outcomes

After delivery, we will record neonatal birth parameters such as the newborn’s sex, birth date, birth weight and length, gestational age, Apgar score, and level of neonatal care required (normal newborn nursery, level 2 or level 3 intensive care). All neonates will be assessed for congenital anomalies immediately after birth. We will condense birth dates into a seasonal scale, classified as cold periods (October to March) and warm periods (April to September).

### Biochemical and Genetic Analyses

#### Blood Sampling

At each visit, we will collect blood samples for epigenetic and genetic analyses and for biochemical analysis. The serum will be divided into 2 aliquots: for routine prenatal tests (fetal blood sampling, cholesterol, low-density lipoprotein, high-density lipoprotein, triglyceride, and insulin), and for measuring 25-hydroxyvitamin D_3_ and bone markers (procollagen I aminoterminal propeptide, osteocalcin, and C-terminal cross-linked telopeptide of type I collagen). The separated sera will be kept at –80°C until analysis.

#### Tissue Biopsy

The tissue biopsy will be taken from the fetal side, 1-1.5 cm below the chorioamniotic membrane at a fixed location in relation to the umbilical cord.

#### Global DNA Methylation Analysis

Genomic DNA will be isolated from placental tissue and the mother’s peripheral venous blood using the standard method.

Briefly, DNA is extracted by the phenol method from whole blood and homogenized placental tissues. We will determine global DNA methylation as previously published [41,42]. Global DNA methylation will be expressed as the percentage of 5-methyldeoxycytidine (5-mdC) versus the sum of 5-mdC and deoxycytidine (dC): [5-mdC/(5-mdC + dC)]%.

#### Gene Expression Analysis

We will extract RNA from peripheral venous blood using a Qiagen kit (QIAGEN NV, Venlo, the Netherlands). Gene expression will be analyzed by using real time polymerase chain reaction after complementary DNA synthesis. Candidate genes include GSH, DNA (cytosine-5)-methyltransferase-1-alpha, SAMe, brain-derived neurotrophic factor, synapsin I, AKT serine/threonine kinase 2, SOS Ras/Rac guanine nucleotide exchange factor 1, SOS Ras/Rac guanine nucleotide exchange factor 2, and phospholipase C gamma 2) in maternal and placental tissues.

### Statistical Analysis

We will present categorical data as frequencies (%) and numbers, and continuous data as mean and standard deviation. We will use chi-square test to compare the prevalence of adverse birth outcomes in the two regions. Student *t* test will compare the differences in global DNA methylation levels in pregnant women in the two regions (polluted and unpolluted) at each trimester.

We will use Spearman correlation coefficients and linear regression to assess the association of global DNA methylation from blood and placental tissue with nitrogen dioxide, PM10, and PM2.5 (data will be obtained from the Air Quality Control Company).

We will construct a stepwise logistic regression model to determine the independent effect sizes of nitrogen dioxide, PM10, and PM2.5 exposures during pregnancy on global methylation. An appropriate cutoff point will be determined for DNA methylation levels, and then the levels will be defined as a dichotomous variable. We will consider covariates for entry into the model, including the newborn’s sex, maternal age (years), gestational age (weeks), parity (1, 2, or 3), sleep duration, dietary intakes of vitamin B_12_ and folate, regional temperature, and season at conception.

The 2^(∆∆Ct)^ formula will be used to calculate relative transcript abundance. Student *t* test will be used to compare gene expression differences of all included genes in blood and placental tissue between the two groups, that is, pregnant women who live in polluted and unpolluted regions. For multiple testing corrections, we will use the false discovery rate [43,44]. We will consider 2-tailed *P* values <.05 as statistically significant. The same analyses will be performed in 2 subgroups from each polluted region: participants with and without classroom education.

## Results

The project was funded in March 2016 and enrollment will be completed in August 2017. Data analysis is under way, and the first results are expected to be submitted for publication in November 2017.

## Discussion

To our knowledge, this is the first birth cohort study in Tehran, which is rated as one of the world’s most polluted cities, to measure global DNA methylation in pregnant women who live in polluted and unpolluted regions and to investigate the interaction between adverse pregnancy outcomes and air pollution as an environmental factor. In addition, we plan to improve women’s knowledge about how to reduce prenatal exposure to air pollution and prevent adverse pregnancy outcomes attributable to air pollutants.

Developmental adaptations due to epigenetic modification may permanently “program” the fetus and may lead to adverse pregnancy outcomes that form the origin of diseases that may arise in adult life.

Based on the evidence, we supposed that prenatal exposures to air pollutants can influence fetal reprogramming by epigenetic modifications such as DNA methylation. This could explain the association between air pollution and adverse pregnancy outcomes.

Of note, there are some potential problems and limitations in our study. Primarily, some confounding factors could have a possible effect on blood and tissue DNA methylation, such as some lifestyle-related factors, environmental tobacco smoke, the season, and environmental temperature. To minimize the impact of lifestyle and regional differences in methylation patterns, we will adjust for the mother’s socioeconomic status, maternal diet, and maternal sleep habits in our analysis. Also, we will consider exposures to other air pollutants such as second-hand smoke and indoor air pollution. We will exclude mothers who smoke or live with a smoker. We will obtain the temperature of each region from Air Quality Control Company data. In addition, the differences in effect estimates of air pollutants on DNA methylation could be further related to differences in maternal nutritional status. To control for the impact of nutritional status, a food frequency questionnaire will be filled out for all participants to measure special nutrients associated with DNA methylation, such as folate and vitamin B_12_. However, we can’t control for some unknown factors that are associated with blood and tissue DNA methylation, as well as levels of air pollutants.

## Abbreviations

GSH: γ-glutamyl-cysteinyl-glycine
PM2.5: particulate matter with particle diameter 2.5 μm
PM10: particulate matter with particle diameter 10 μm
SAMe: S-adenosylmethionine
WHO: World Health Organization
